# Experiences of HIV-positive postpartum women and health workers involved with community-based antiretroviral therapy adherence clubs in Cape Town, South Africa

**DOI:** 10.1186/s12889-018-5836-4

**Published:** 2018-07-31

**Authors:** Zara Trafford, Yolanda Gomba, Christopher J. Colvin, Victoria O. Iyun, Tamsin K. Phillips, Kirsty Brittain, Landon Myer, Elaine J. Abrams, Allison Zerbe

**Affiliations:** 1 Division of Social and Behavioural Sciences, School of Public Health and Family Medicine, University of Cape Town, Cape Town, South Africa; 2 Centre for Infectious Disease Epidemiology and Research, School of Public Health and Family Medicine, University of Cape Town, Cape Town, South Africa; 3 Division of Epidemiology and Biostatistics, School of Public Health and Family Medicine, University of Cape Town, Cape Town, South Africa; 4 Mailman School of Public Health, ICAP, Columbia University, New York, USA; 5 College of Physicians and Surgeons, Columbia University, New York, USA

**Keywords:** Differentiated care, Adherence clubs, Postpartum women, Health workers, Antiretroviral therapy, HIV, South Africa

## Abstract

**Background:**

The rollout of universal, lifelong treatment for all HIV-positive pregnant and breastfeeding women (“Option B+”) has rapidly increased the number of women initiating antiretroviral treatment (ART) and requiring ART care postpartum. In a pilot project in South Africa, eligible postpartum women were offered the choice of referral to the standard of care, a local primary health care clinic, or a community-based model of differentiated ART services, the adherence club (AC). ACs have typically enrolled only non-pregnant and non-postpartum adults; postpartum women had not previously been referred directly from antenatal care. There is little evidence regarding postpartum women’s preferences for and experiences of differentiated models of care, or the capacity of this particular model to cater to their specific needs. This qualitative paper reports on feedback from both postpartum women and health workers who care for them on their respective experiences of the AC.

**Methods:**

One-on-one in-depth qualitative interviews were conducted with 19 (23%) of the 84 postpartum women who selected the AC and were retained at approximately 12 months postpartum, and 9 health workers who staff the AC. Data were transcribed and thematically analysed using NVivo 11.

**Results:**

Postpartum women’s inclusion in the AC was acceptable for both participants and health workers. Health workers were welcoming of postpartum women but expressed concerns about prospects for longer term adherence and retention, and raised logistical issues they felt might compromise trust with AC members in general.

**Conclusions:**

Enrolling postpartum women in mixed groups with the general adult population is feasible and acceptable. Preliminary recommendations are offered and may assist in supporting the specific needs of postpartum women transitioning from antenatal ART care.

**Trial registration:**

Number NCT02417675 clinicaltrials.gov/ct2/show/record/NCT02417675 (*retrospective reg.*)

## Background

The last two decades have brought major advances in the prevention of mother-to-child transmission (PMTCT) of HIV [1]. With the introduction of universal, lifelong antiretroviral therapy (ART) for all pregnant and breastfeeding women known as “Option B+”, the number of HIV-positive women initiating ART has also increased dramatically [2]. However, these advances are simultaneous with broader concerns that ART adherence and retention in HIV care among HIV-positive women may be suboptimal, particularly during the postpartum period [3–5]. These issues are even more acute in sub-Saharan Africa, where limited resources and a large patient population pose additional challenges [6, 7]. To address some of these concerns, there has been a shift towards the integration of ART into routine antenatal care, rather than providing ART services at a separate location during pregnancy [8]. However, providing ART and HIV care postnatally remains a significant challenge, and there is little evidence about strategies for the optimal delivery of these critical services during the postpartum period, or about which models of care would be most acceptable for this population [9].

In an attempt to treat all people living with HIV, ART coverage for the general population has also expanded rapidly in recent years [10]. As this coverage has increased, health systems have struggled to support an ever-growing population of patients enrolled in ART care. In response, innovative models of differentiated care, including clinic-based medication adherence clubs (MACs) and community adherence groups (CAGs), have emerged [11, 12]. Pioneered in the Cape Town Metro in South Africa, the community-based adherence club (AC) is another model of differentiated care, which aims to help decongest ART clinics by shifting stable, non-pregnant patients to community-based services [13]. MACs, CAGs and ACs are innovative models of care adopting slightly different applications of the same general principle: decentralised, differentiated care services for the distribution of medication, which are sometimes combined with educational and social support. Once HIV-positive patients are adherent, report no comorbidities, and have a suppressed viral load, they can be referred out of local primary health care clinics (PHCs) into a network of community-based ACs. In contrast to the PHCs where ART is usually distributed, ACs have usually been situated outside clinics or other health facilities and require less frequent visits because multiple months of medication are distributed at each visit [14]. An itemised distinction between the two is represented in Table 1 [15]. This club system has yielded promising results, both in terms of boosting retention and decreasing the burden on an overloaded public sector health system [12, 16, 17].

**Table 1:** Comparison of key features of primary care clinics and adherence clubs [15]

Category	Primary Care Clinic (PHC)	Community-based Adherence Club (AC)
Setting	Clinic-based	Community-based
Patient profile	All ART patients	Stable patients
Key personnel	Doctors/nurses	Lay counselors
Frequency of visits	1–2 monthly	2–4 monthly
Frequency of clinical consultations	1–2 monthly (every visit)	12 -monthly
Emphasis of patient contacts	Detecting clinical complications	Treatment adherence, patient wellness
Units of care	Individual patient	Groups of 25–30 patients
Peer-based support	No emphasis	Strong emphasis
Patient self-management	Minimal emphasis	Strong emphasis
Frequency of laboratory monitoring for stable patients	12-monthly	12-monthly
Management of clinical complications	On-site	Up-referral to PHC
ART packing and dispensing	Packed at the clinic pharmacy, dispensed from pharmacy	Pre-packed by central dispensing unit, dispensed at AC visit
Treatment “buddy”	Patients must collect ART themselves	ART can be collected by a treatment “buddy”

Previously, the AC model focused exclusively on non-pregnant adults with no attention to whether and how the AC approach may be extended to include women during the postpartum period. With the roll-out of Option B+, the AC model may offer a viable and attractive alternative for the large numbers of HIV-positive postpartum women who are or will be exiting integrated ART and antenatal services and need to be absorbed into routine ART services. Recent World Health Organization (WHO) guidelines and a decision framework produced by the International AIDS Society (IAS) support the careful introduction of key and higher-risk populations into services for differentiated care as long as clinical and viral monitoring is maintained [18, 19]. However, although the emerging evidence indicates comparable clinical outcomes in differentiated versus standard systems of ART distribution and care, there is little evidence regarding postpartum women’s *preferences* for models of care, their *experiences* of differentiated care, and the *capacity of this model (the AC)* to cater to their *specific needs* [20].

As part of a large implementation science study examining models of care for HIV-positive pregnant and postpartum women, we conducted a pilot study in which newly postpartum, breastfeeding women who initiated ART in pregnancy and met local criteria for club membership were offered the choice of referral to their local ART clinic or to an existing AC in Gugulethu, Cape Town for ongoing HIV and ART care. In line with international guidelines, local criteria for club membership eligibility stipulate that the HIV-positive individual should be virally suppressed (HIV RNA < 1000 copies/mL) per most recent viral load test, clinically stable with no active co-morbidity or opportunistic infections, and currently resident in a catchment area appropriate for referral to an AC [21]. This paper presents the findings from the qualitative component of the study, which was designed to elicit feedback from both postpartum women and health workers who care for them on their respective experiences of the AC.

## Methods

A sub-study to the NIH-funded Maternal and Child Health-Antiretroviral Therapy (MCH-ART) implementation science study (NCT01933477) [22], the Postpartum Adherence Clubs to Enhance Support (PACER) study (NCT02417675) aimed to explore the acceptability and feasibility of referring HIV-positive postpartum women on ART into an existing network of community-based ACs, a local model operating in Cape Town since 2012 under public sector provisions [23].

**Fig. 1:**
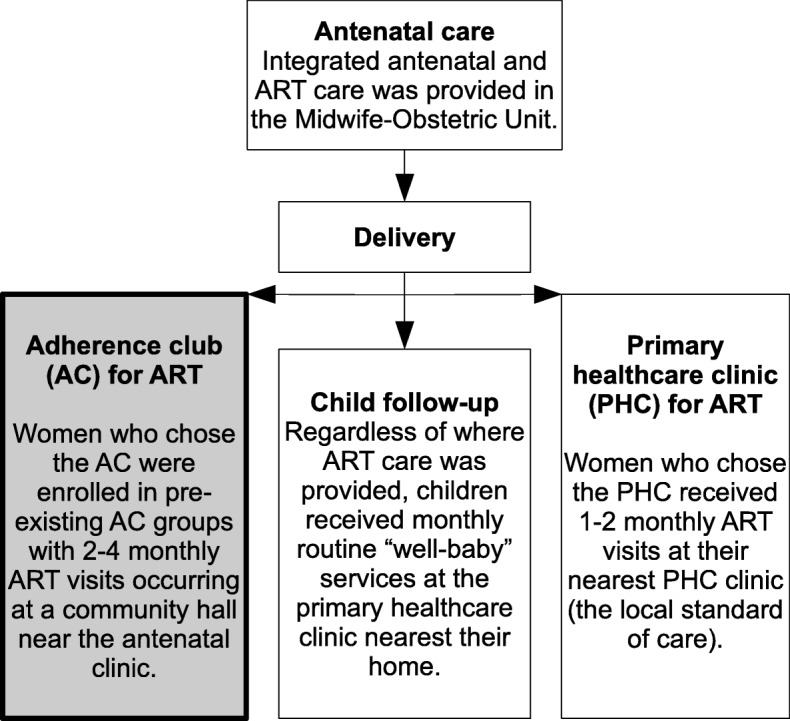
Receipt of care from antenatal to postpartum services

Newly postpartum breastfeeding^1^ women who initiated ART in pregnancy at the same Midwife-Obstetrics Unit and who had achieved viral suppression were consecutively enrolled and offered a choice between the AC and a local PHC for postpartum ART care. Women who selected the AC were enrolled in pre-existing AC groups at approximately the same time as the rest of the cohort enrolled at PHCs. In both systems, postpartum viral load was measured annually and routine infant care was provided separately through a network of “well-baby” clinics (Fig. 1). In the PHC, visits are 1–2 monthly and include medication collection and a clinical consultation with either a doctor or nurse at every visit.

In comparison, within the AC system, standard visits with medication collection are 2–4 monthly and are facilitated by lay counsellors, known locally as community health workers (CHWs). Nurses from the local facility rotate through club sessions and using an appointments system, provide one annual blood test and one annual clinical consultation for each member. Club members do not receive other medical care at clubs; if they present with symptoms requiring clinical attention they are referred to a local clinic. At each visit patients are weighed and CHWs deliver group health education talks. ART can be collected by a treatment “buddy” every second visit, as long as this does not coincide with their clinical review or blood test. All women enrolled in the PACER study were followed in the quantitative sub-study through 12 months postpartum, with study measurement visits occurring separately from AC or PHC visits for ART care [21]. Key findings include (i) the wide range of women’s reasons for choosing ACs versus PHCs, and (ii) comparable virologic and retention outcomes in women choosing ACs versus those choosing PHCs [21]. Additional details related to the design and preliminary quantitative results of this study are also available [21].

This paper reports on the qualitative component of the PACER study. We conducted one-on-one in-depth interviews with a sample of the women who chose to receive postpartum ART care from an AC. Women who were engaged in care at 12 months postpartum were invited to participate in interviews and were approached sequentially, according to their next scheduled AC appointment date. We stopped recruitment once we had reached our intended sample size. Those who selected PHCs were not approached or interviewed for this component of the study.

In addition, one-on-one interviews were conducted with nine health workers involved in providing HIV services to AC members, including 3 local facility nurses and 6 CHWs. These health workers were already actively providing services and were not specifically hired or trained for this study. The health workers interviewed represent all of the CHWs and 3 of the 6 nurses (based on a convenience sample) involved with the Gugulethu ACs at the time of the study. Health workers were recruited by the study coordinator. Many of these heath workers have years of experience in ART services and have been exposed to a wide variety of patients and models of care; their insiders’ view on both the AC and the clinic system adds an additional layer of insight to our understanding.

Data were collected from July to September 2016. Interviews took place in a private room at the study research office at the Gugulethu community health centre (CHC). All interviewees signed informed consent forms which were explained verbally and had been translated into isiXhosa. Interviews were conducted by a trained, first-language isiXhosa-speaking research assistant using a semi-structured interview guide. Interviews were audio-recorded, translated, and transcribed verbatim. Our inductive approach to data analysis entailed regular analysis meetings between the core qualitative research team (involving the research assistant, primary author, and senior qualitative researcher), which facilitated discussion and refinement of emerging findings. We used NVivo11 to code transcripts, identify themes, and recode transcripts to highlight focal areas. No signifiers are provided for the quotes below as anonymity was assured and the group was similar in terms of demographic characteristics.

The study received ethical approval from the University of Cape Town Faculty of Health Sciences Human Research Ethics Committee (HREC) and the Columbia University Medical Center Institutional Review Board (IRB).

## Results

Of the total cohort of 129 HIV-positive postpartum women enrolled into the PACER study, 84 (65%) chose to join an AC for ART care and the remaining 45 (35%) selected PHCs, with no demographic or clinical predictors of this choice [21]. In-depth interviews were conducted with 19 of the women who had selected the AC, approximately 23% of those who elected to join the AC. At the time of interview, women had attended a median of 5 AC visits (IQR, 5–7). All 19 IDI participants were engaged in care at 12 months postpartum, compared to 55 (85%) of the 65 women who chose clubs and were not interviewed. Just under half (9; 47%) of the interviewed group had been diagnosed with HIV during their most recent pregnancy. Previous use of antiretroviral drugs (ARVs) for PMTCT was uncommon for most of the group and only 32% of women had a history of any ARV use prior to their current pregnancy. Women were referred out of antenatal care and into ACs at a median of 7 days post-delivery (IQR, 5–21) after a median of 21 weeks on ART (IQR, 18–25). Additional demographic and clinical details are provided in Table 2. There were no appreciable demographic differences between the in-depth interview participants and the broader group of women who chose ACs.

**Table 2:** Demographic and clinical characteristics of postpartum women attending adherence clubs (ACs) and participating in in-depth interviews^a^

Variable	Participants choosing ACs – n (%)	In-depth interview participants – n (%)
Number of participants	84	19
Median [IQR] age in years	29 [24, 31]	29 [23, 33]
< 25 years	22 (26)	6 (32)
25–34 years	50 (60)	9 (47)
≥ 35 years	12 (14)	4 (21)
Home language	82 (98)	18 (95)
isiXhosa	2 (2)	1 (5)
Other		
Educational attainment		
Less than secondary	42 (50)	9 (47)
Completed secondary/any tertiary	42 (50)	10 (53)
Employment status		
Not employed	51 (61)	11 (58)
Currently employed	32 (38)	8 (42)
Relationship status		
Married/cohabiting	32 (38)	7 (37)
Single	52 (62)	12 (63)
Parity		
Primaparous	18 (21)	3 (16)
Multiparous	66 (79)	16 (84)
Pregnancy intention		
Unintended	59 (70)	12 (63)
Intended	25 (30)	7 (37)
Newly diagnosed HIV+ in pregnancy		
Newly diagnosed	48 (57)	9 (47)
Diagnosed previously	36 (43)	10 (53)
Any ARV use before pregnancy		
No previous ARV use	58 (69)	13 (68)
Previous use of ARVs for PMTCT only	17 (20)	4 (21)
Previous use of ART	9 (11)	2 (11)
CD4 cell count nearest referral to AC		
≤ 350 cells/μL	34 (42)	8 (42)
> 350 cells/μL	47 (58)	11 (58)
HIV viral load nearest referral to AC (*n* = 80)		
< 50 copies/mL	74 (93)	15 (88)
50–999 copies/mL	5 (6)	2 (12)
≥ 1000 copies/mL	1 (1)	0 (0)
Median [IQR] days postpartum at referral to AC	9 [5, 21]	7 [5, 21]
Median [IQR] weeks on ART at referral to AC	23 [19, 27]	21 [18, 25]

^a^All cells are n (%) unless specified otherwise

Eight of the nine health worker interviewees had been working with ACs from around the time of inception (approximately 4 years at the time of interview) and only one of the health workers was male. The quotes from healthcare workers in the findings below are differentiated using the code “N” or “CHW”, denoting comments made by a nurse or a CHW respectively.

### Postpartum women’s perspectives on the AC system

#### Overall acceptability

The club system was highly acceptable to the postpartum women interviewed in this study. Participants cited the benefits of less frequent appointments, short waiting times and no queues, and better care and service than they had experienced at public clinics.

*“…it suits me. You stay without stress for 2 months. You sit and relax knowing that next month you will go and fetch your tablets.”*

*“…at the clinic [for other medical issues], you stay the whole day. It is full and [often] you don’t even get [medication].”*

*“…the environment [at the AC] is more… interactive. At [the PHC], when you are finally attended to, the person who attends to you is already tired.”*

Some reported unhappiness at being referred to a clinic when ill instead of being able to access care at the AC, explaining that, “if you are sick [or in pain]… you do not get time to talk [about the illness].” However, most women were glad that they and their infants were protected from direct and extended contact with “sick people” and could avoid long, regular visits to health facilities. In addition, participants appreciated the fact that the club was not identifiable as an ART distribution point, which they felt helped in avoiding the stigma associated with collecting medication from clearly demarcated areas.

*“We know… that a person who enters at [the PHC] takes HIV treatment… I told myself that [if I ever found myself] in the same situation as [that] person, I would rather not eat medication. I never knew that there were options like a club… quick, quick and you leave.”*

Most participants welcomed the opportunity to be around people who had the same diagnosis. Women commented on the benefits of sharing their experiences of taking medication, getting helpful tips from long-term AC members, and most importantly, seeing HIV-positive peers looking and feeling happy and healthy, which gave them courage and comfort.

*“The people who are there have one disease, we speak the same language… I [discovered] that others have been living with this virus for 15 years. When we talk there, we listen to one another. You realise that this [person has been living with HIV] for some time but she still looks beautiful.”*

*“Now I [have] people who know me. We are like a family… We take each other as brothers and sisters.”*

A few postpartum women felt intimidated by their peers, explaining that they felt too shy to ask questions in a group setting and would have preferred one-on-one counselling. Two young women also described having felt judged by other older female participants.

*“I don’t like talking, especially in front of too many people.”*

*“If they want to hear people’s problems they should listen to them one by one… if they listen to [us] talk in front of others, others will say, ‘No!’ [They will think], ‘Wow, this child is too young!’. Most of the people [in my club] are older people. [There are few of us] young ones.”*

*“I am not free to talk [in front of my elders]. I am just not free.”*

Finally, though not a major concern, some women expressed confusion because they received clinical reviews less frequently at the AC (annually) than during antenatal care (1–2 monthly), which entailed more regular and intensive clinical evaluations to monitor the pregnancy and prevent mother-to-child transmission of HIV. These women were concerned that their CD4 and viral load measurements might not have been properly managed while attending ACs.

#### Interaction with routine infant health service visits

Postpartum women take their infants for monthly “well-baby” check-ups at a separate facility, raising the possibility that multiple health visits to different locations might be onerous and could prevent AC attendance. The women we interviewed did not mind taking their baby to regular infant health check-ups in a different location on top of attending the AC. Some reported that when their AC and “well-baby” dates had clashed in the past, they had chosen to attend the AC first (because it was fast) before traveling to the baby’s appointment. They were comfortable with the separation of services because the AC was efficient and their infants were not HIV-positive.

*“[The club] is nearer than [the baby clinic] so even if I have an appointment [at the baby clinic], I go to [the club] first and then take my card to [the baby clinic]. I easily juggle between the two.”*

#### Views on club health workers and health education sessions

Postpartum women were positive about health workers at the AC and were especially grateful that health workers could be contacted directly. Participants appreciated health workers’ attentive care and contrasted this with upsetting interactions on previous visits to health facilities. Participants also appreciated the flexibility at the club; latecomers were gently reprimanded but were usually given the opportunity to come late, receive a new club date, or send a friend to pick up medication on their behalf.

*“[CHWs at the AC] are very kind. They care a lot.”*

*“[CHWs at the AC are] friendly, like they do not belong to the clinic... Nurses [at the clinic] are harsh”*

*“They advise us not to be late. They speak nicely [and] don’t scold us.”*

While it did not emerge as a prominent theme, one participant noted that health workers had low expectations of postpartum women transferring from antenatal care and that some had explicitly said that they did not expect these women to be retained in the AC. This particular woman was motivated by these low expectations but felt that other postpartum women might be dissuaded from coming back if this was their first impression of the AC. Quoting a health worker, she said:

*“‘We know [the women referred from antenatal care]. They only come once and you will never see them again.’ I told myself that I will show these ones that I care about my health… I will show them that I came to the club to stay.”*

In addition to weighing club members and distributing medication, CHWs also deliver group education sessions. Most postpartum women interviewed had had some exposure to health education but both participants and health workers indicated that the depth, scope, and impact of these sessions were extremely variable. The women who remembered educational sessions valued the opportunity to learn and to discuss their concerns. Some reported that they had not received any health information at all but acknowledged that they may have missed sessions due to lateness, newness in the system, or rushing to get to work.

*“They advise us on the way we must take treatment [and] about the time… ‘If you take it at 9h00, you must know that you stick to 9h00’. Secondly, they advise us to use a condom… and not to stop because [then] you let the virus spread. [Another] thing [is] alcohol… Taking treatment and alcohol is like playing [with your life].”*

*“What I like [about the AC] is that we are being taught about this disease we have… We are educated.”*

While the primary purpose of the AC education sessions is to improve treatment literacy, some participants also reported having received condoms and additional information about contraception, health screening, and the management of other diseases or conditions. Very few women reported receiving maternal and child health (MCH)-related information (e.g. infant-feeding guidance). The latter seems to have been provided occasionally but in an ad hoc fashion by rotating nurses, rather than by CHWs during the health education group talks.

*“Most of the people there don’t have babies. I get the chance to ask the [nurse] about things when I go [to be weighed]. They don’t normally stand in front of us and address issues regarding babies.”*

*“I haven’t [been given any infant-care advice at the club] since I was told [during antenatal care] how to feed a baby.”*

#### Diagnosis and disclosure

Despite the uneven penetration of health education sessions, many participants emphasized the importance of coming to terms with their diagnosis and good treatment literacy, which some credited to their involvement in the club. Some explained that disclosure had also helped facilitate their adherence.

*“At first… I thought it was going to change everything. But it didn’t. I [understood] after they told me everything – if you are taking medication, you will stay healthy. You use protection and control your CD4 count. There is no… sign on your forehead that shows that you are positive.”*

*“Something I found helpful is to tell your family and… your boyfriend... It’s a good life when your family knows. HIV is not death. What is important is to… follow the procedures.”*

In contrast, some women reported low disclosure rates and some had not yet disclosed to their partner. All of the women also talked about the effects of stigma on their ability to adhere; even those who emphasised the importance of disclosure were concerned about how they would be perceived by colleagues, family, or the broader community should their status be revealed.

*“Sometimes… I work night shift [but] because I [haven’t] told them… they don’t know that I am eating treatment. So it [is] difficult to take my pill during the times that I’m supposed to… I fear the way they will behave around me when they know that I am positive.”*

### Health worker perspectives on the enrolment of postpartum women in the AC system

#### Overall acceptability of the AC

Both CHWs and nurses expressed support for the AC system and a belief that ACs provide an efficient, effective and caring service, as well as alleviating pressure on the health system.

*“If those people were not in the club, they would have been [at the PHC]. People from [a] hundred clubs would come to overcrowd the clinic. It [has made] a difference, although the clinic is still full.” (CHW)*

*“[The AC] makes their lives easier. They can go back to work [more quickly].” (CHW)*

Although mostly satisfied, health workers identified logistical problems including a lack of dedicated transport to the AC location and the regular unavailability of medication, attributing the latter to pharmacy issues including miscommunication and a problematic supply and delivery chain. Health workers found these issues personally distressing and believed they eroded club members’ trust.

*“We are late because of the transport; sometimes there is no driver or there is no car… [and] when we arrive at the club, we find them angry.” (CHW)*

*“When people come to fetch their medication and don’t get it, they have to go to the clinic – that makes people angry.” (CHW)*

*“Clients have to walk long distances to get their medication which is risky because… that put[s] clients at the risk of being robbed.” (CHW)*

#### Postpartum women, health information, and the transition from antenatal care to AC

While CHWs and nurses both welcomed the introduction of postpartum women into the AC, they had low expectations for the retention of women who were referred from antenatal care.

*“The only people we sometimes have problems with are people who come from [antenatal care] that are referred to the club. Sometimes they don’t keep their appointments.” (CHW)*

*“Most women who just gave birth are defaulters… they were defaulters previous[ly] and they still are… Some newer ones are compliant but previous defaulters do the same thing at the club.” (N)*

This differed from their positive opinions of other (non-postpartum) club members who had first received ART through PHCs but had now been part of the AC for some time.

*“It is easy to work with them because they are well-informed. They know what the programme is about, they know about their sickness, they [know about] the benefits of taking treatment. They know the importance of adhering to treatment. They know the side-effects. They know about disclosure. They are more informed than people at the clinic, especially new [ART initiates].” (N)*

Health workers hypothesised that the relatively low prospects for adherence and retention might be linked to youth and forgetfulness, an overlap with other appointments for their children, or incomplete disclosure.

*“[Young women] are forgetful, they always miss their dates” (CHW)*

*“I assume that they are used to being called. We do not call them, we just encourage them to keep their appointments… [or] maybe they find out that their child is booked on the same date as the club and forget to call the club.” (CHW)*

*“You [often] find that for young women, the problem will be disclosure.” (N)*

In addition, nurses and CHWs both commented on what *they* saw as a key difference between women who had been referred directly from antenatal care and the other adults in the club: the latter had only been referred after their completion of treatment readiness classes and demonstration of longer-term adherence and stability.

*“When they go straight from [antenatal care] to the club, you are not sure about what happens to her.” (N)*

*“Those who come from [antenatal care] aren’t like those who come from [PHCs]. You need to educate them more because [in the PHC], there are classes.” (CHW)*

*“…[they don’t] have experience… Those who start [treatment at the clinic] go through those [initiating] classes. They are drilled and drilled… about treatment [and] about positive living and adherence… Now [women in antenatal care go to the club] raw, raw, raw…” (N)*

CHWs felt that postpartum women would benefit from more targeted attention but some felt insecure about providing guidance because they felt their information about ART was outdated. They tended to share the information they were most familiar with and this varied according to the CHW in question, potentially resulting in cumulative gaps in critical information for members.

*“We need to get trained because [our last training was] long ago. I sometimes equip myself by going to HIV support group sessions [in my personal capacity] – that’s where I get information.” (CHW)*

*“I usually talk about what I know and will be able to deal with.” (CHW)*

*“We choose a certain topic. We don’t say everything, otherwise we will bombard them with information they are going to forget.” (N)*

Finally, similarly to study participants, CHWs and nurses generally reported that targeted MCH information was not routinely provided at the AC. While nurses were sometimes able to provide this input due to previous midwifery or related training, most CHWs had not received training in this area and felt unprepared to present this information. Some also felt that information sessions should be broad and not specific to one target group because the general population at the AC comprises mixed ages and genders.

*“We don’t direct topics to a specific group of people… we choose general topics that affect all our clients irrespective of gender.” (CHW)*

*“We don’t normally emphasise mothers who just gave birth, we don’t normally have them [in the AC].” (CHW)*

*“We need more skills, especially with regards to breastfeeding… we still need [MCH] training because we would love to inform them about everything, but we don’t have enough information.” (CHW)*

However, in light of the challenges of new motherhood and the postpartum period, others felt strongly that it was important to cater to these women by incorporating MCH-related information into provisions at the club.

*“We want them to come, and then this and that is done, and they must leave. Young mothers need more… [not only collecting pills] and then leaving. Sometimes they need to be sat down and asked if they [are coping] with the child. But [that] wasn’t in the planning [for the AC], that there would be those mothers with [infants] who still need to be asked about their baby.” (N)*

*“Some claim they never heard of the information you are telling them about. They need to be advised on how to feed their babies; those who breastfeed need to be told not to mix breast milk with the formula.” (CHW)*

## Discussion

Evidence indicates that ACs work well for stable, non-pregnant adults but less is known about whether this model can be extended to the growing population of postpartum women on ART. The PACER study explored the feasibility of referring postpartum women directly from antenatal care to an AC, while this qualitative component was designed to better understand how these clubs operate by exploring the experiences of some of these women and of the health workers who staff the ACs. In this section, we expand on common interesting themes that emerged and offer preliminary suggestions for minor changes to enhance AC service provisions for this specific group.

It was clear that participation of postpartum women in ACs was acceptable for both participants and health workers. The women we interviewed appreciated the service and particularly noted the attentive care they had received from AC health workers. The benefits cited focused primarily on shorter waiting times, fewer regular visits, and the opportunity to access services outside health facilities. These advantages have also been reported in other literature on differentiated care for the general adult population [17, 24, 25]. Health workers considered the service acceptable and reported promising health outcomes, positive reactions from their clients, and the decongestion of clinics and other health facilities. Health workers also described logistical challenges they had faced and explained that these might cause breakdowns in trust and in the longer-term, potentially degrade the acceptability of the AC.

In addition to these more generic health systems issues, there were also important findings relating to postpartum women. For example, there was concern that low health worker expectations of postpartum women’s likelihood of success could complicate the relationship between health workers and postpartum women. A trusting relationship between health workers and patients has been shown to be a key facilitator of improved access and adherence to treatment among pregnant and postpartum HIV-positive women [26–28]. Considering the introduction of Option B+ and the likely surge of new ART initiates who are also in the postpartum phase, it is important to try and ensure that as they enter the AC these women are not treated with prejudice because of assumptions about poorer adherence and retention outcomes.

Health workers said that low expectations for postpartum women’s retention and adherence stemmed, in part, from a perceived lack of treatment literacy and preparation for adherence among those postpartum women referred directly from antenatal care. One health worker described these women as “raw”. Other (neither pregnant nor postpartum adult) AC members are eligible for AC membership once they have demonstrated stability and adherence at a PHC clinic for at least 6–12 months [21]. In addition, people living with HIV (PLWHIV) and accessing local standard of care services have typically been expected to take a series of treatment literacy and adherence classes prior to initiating ART. Women in the PACER study were referred out of antenatal care and into the AC system slightly earlier, just under 6 months after initiating treatment. They had received counselling about PMTCT, adherence, and infant feeding practices but this was provided concurrently with antenatal care; there was no specific ART initiation programme for the postpartum women who transferred to ACs. This was an intentional choice because the study aimed to work within the existing structures of the local health system to explore the feasibility of directing newly postpartum women into the system as it is currently operating, rather than creating new or additional interventions [21].

Due to their vulnerability and higher risk profile, pregnant and postpartum women may require additional support [18, 19]. For example, the majority of the women we interviewed were newly initiating ART, almost half were also newly diagnosed, and incomplete or non-disclosure was widely reported. These factors have been linked to default and disengagement from care [28, 29]. While this vulnerability is likely to be applicable to all postpartum women who might be referred to *any* service for the distribution of ART, the AC requires a higher degree of self-management and longer periods without contact with health workers. In addition, as this was a pilot project to examine feasibility, it is important to consider and address areas that might be easily strengthened at an early stage, before they become more significant problems.

### Initial recommendations for enhancing AC service provision for postpartum women

#### Bridging preparation

Some of the women who were interviewed seemed well-versed in ART and accurately relayed how ART should be used. Others did not seem to be getting enough exposure to information and some were wholly unaware of the educational talks provided by health workers. In addition, health workers expressed particular concerns about the “rawness” of postpartum women and potential gaps in treatment literacy. Effective treatment literacy and self-empowerment have previously been established as valuable facilitators of adherence [30–33]. Recent WHO and IAS guidelines have reiterated the sensitivity of the immediate postpartum period [18, 19]. Although referral directly from antenatal care is clearly possible (as evidenced by this group’s successful transition), the transfer might be eased by the introduction of bridging activities to support treatment literacy for postpartum women. Such activities could moderate the confusion some of the women transferring between antenatal and adult ART care felt when the regularity of clinical reviews decreased, and assist in preventing MTCT. For example, women could be provided with a simple schema tabulating the differences between the two services, which could then be verbally reiterated during this “bridging” period. Regular repetition of health education is already built into the club format through CHWs’ sessions but this critical information is likely to be more consistently absorbed if it builds on a stronger foundation, particularly for women already dealing with additional new responsibilities in the early postpartum period.

#### Lay health worker training and incorporation of targeted MCH information

CHWs who were interviewed expressed insecurity about their overall knowledge, as well as their access to training and up-to-date ART guidelines. Increased task-shifting and the rapid growth of the HIV-positive population engaged in care and receiving treatment have brought lay health workers to the forefront of ART distribution and care (particularly in sub-Saharan Africa) and their delivery of HIV testing, counselling and ART has been found to be both efficient and effective [20, 34]. In their review of Cape Town AC outcomes in particular, Tsondai et al. describe high rates of retention and viral suppression but emphasise that the quality of care must be maintained as programmes for the delivery of ART are scaled up [35]. Indeed, inadequate training of lay health workers has been specifically identified as a key barrier to adherence [27] and conversely, there is strong evidence in favour of up to date, repeated training [27, 36–38]. The outdated and insufficient health worker training highlighted by CHWs could thus pose a threat to sustainability and dilute the longer-term positive effects of the AC for this high-risk group. We recommend that CHWs are regularly provided with refresher training for ART literacy as part of an ongoing programme of support that is required for lay health workers to effectively manage ACs.

Additionally, CHWs working in ACs are not routinely trained to provide MCH information. In debates about differentiated ART care, there has been speculation about the advantages and disadvantages of setting up customised models of differentiated care for specific populations [19]. For postpartum women, this kind of targeted group might include services such as family planning and infant feeding counselling. However, there have been pragmatic objections that PHCs would be redundant if mechanisms for specific primary care functions are simply reproduced in differentiated care mechanisms, and that this could increase complexity and expense within already overburdened health systems [21]. While it is difficult to predict the outcomes of an MCH-focused customisation, interview results indicated that participants did not consider MCH information or services a significant unmet need and women were generally satisfied with (or even preferred) the separation of infant health and wellbeing services from ART distribution. However, health workers felt that the lack of MCH information was a significant gap and emphasised the need to cater for these women within the AC system, especially if postpartum women were to be enrolled on a larger scale. Although customised groups may be unnecessary, emerging evidence shows that as HIV science shifts and expands, the need for complementary additional counselling is becoming increasingly apparent [39]. There is scope for providing CHWs with some training and relevant MCH information so that they feel adequately equipped to offer guidance to this new population with its specific needs. Referral networks to nearby psychosocial support services should also be strengthened and widely publicised to all members of the AC, especially postpartum women [19].

#### Additional support through counselling and reminders

Results indicated that although the club helped to form supportive relationships, some women in our sample were shy and others felt they would cross cultural generational boundaries by expressing themselves in front of older club members. Improved adherence has been linked with interventions that are socio-culturally acceptable and build empowerment through the establishment of strong and supportive social relationships between PLWHIV [27]. Furthermore, the routing of higher risk populations into differentiated care must be reinforced with appropriate psychosocial support through peer counselling or properly trained lay health workers [18, 19]. In situating the AC outside of clinics and implicitly acknowledging the impact of stigma, this model of differentiated care was responsively designed for its social context. However, some expressed difficulties with the sociocultural dynamics around age differences in the group, which suggests that designers of differentiated care interventions might also consider developing accompanying CHW training materials or information sessions that encourage sensitivity to the silencing or stigmatising effects of generational dynamics. Women were generally positive about CHWs at the AC, which suggests they may respond well to more explicit offers of one-on-one counselling or the opportunity to ask questions in private with these caring health workers.

Finally, most women in PACER interviews commented on the considerable value of regular telephonic reminders they had received during their prior antenatal care, and the occasional ad hoc SMS reminder they received from CHWs at the club. Health workers also believed (young) postpartum women were “forgetful” and that this was one of the main reasons for their disengagement from care. Although the data are highly mixed, phone-based interventions have in some studies appeared to be associated with improved early postpartum retention among women in PMTCT care [9]. Notwithstanding the complexity of delivering mHealth interventions, it is possible that for the first year of membership in the club, a simple automated reminder system could boost postpartum women’s attendance at the AC.

### Limitations

The study was limited in its generalisability. The Discussion only comments on the experiences of those who selected the AC and were retained in care to approximately 12 months postpartum. It is important to remember that most of these women had recently initiated ART for the first time and any prior exposure to ART care was through antenatal check-up and maintenance visits, which were more regular and intensive. They had not experienced routine services for ART distribution and care. The potential challenges for this group of postpartum women may thus be similar or even more severe for women moving from antenatal care into *any system* of adult ART care. We were also unable to compare the experiences of this group of women and the rest of the cohort, as we only interviewed a sample of women who had chosen the AC over PHC for receipt of ART. Finally, we could not describe longitudinal shifts in attitudes and treatment literacy as interviews were once-off. Despite these constraints, we believe our findings offer a useful starting point and considerations for future interventions to provide ART to this rapidly-growing population. We hope that the preliminary insights from this exploratory study will assist in the thoughtful design of future interventions experimenting with differentiated care for high risk adult populations.

## Conclusions

Postpartum women were effectively absorbed into the AC system and the sample we interviewed at approximately 12 months postpartum found their experiences to be generally positive and beneficial. Postpartum women’s enrolment in ACs was also acceptable for health workers, although the concerns they expressed highlighted areas in which provision through the AC could be strengthened for postpartum women. Based on existing evidence, the system seems well-designed to facilitate adherence and retention in care, although there are opportunities for improving the sustainability, reach, and efficacy of facilitatory factors for this specific group.

This qualitative study has provided preliminary insights about the experiences of HIV-positive postpartum women and health workers involved with community-based antiretroviral therapy adherence clubs in Cape Town, South Africa. These insights are context-specific but may offer initial directions for making the AC welcoming for postpartum women with the introduction of relatively minor enhancements. As the AC system is already functional and established in the city, this offers an attractive opportunity to alleviate the rapidly increasing caseload in the health system signalled by the shift to Option B+, without relying on additional funding or new interventions.

Further research with varied samples of postpartum women is required in order to better understand how to optimize the critical transfer between antenatal and adult ART care in different contexts, and to develop feasible and acceptable care delivery models for the delivery of lifelong ART to postpartum women. Longitudinal research is also essential to monitor outcomes and to improve our understanding of the impact of AC membership on postpartum women’s adherence and retention in care. Research in other settings would highlight context-specific issues and long-term quantitative clinical outcomes should also be tracked. We affirm the need for additional work to explore these varying experiences of differentiated care, and to try and better understand HIV-positive postpartum women’s movement through the treatment cascade. This paper offers initial suggestions to inform future research and intervention.

## Abbreviations

AC: Adherence club
ART: Antiretroviral treatment
ARV(s): Antiretroviral(s)
CHC: Community health centre
CHW: Community health worker
MCH: Maternal and child health
MCH-ART: Maternal and Child Health-Antiretroviral Therapy (implementation science study)
PACER: Postpartum Adherence Clubs to Enhance Support (mixed methods study)
PHC: Primary health clinic
PLWHIV: People/person living with HIV
PMTCT: Prevention of mother-to-child transmission (as related to HIV)

## Footnotes

1 Due to the continued risk of MTCT during breastfeeding, one of the sub-objectives of the core quantitative study was to investigate maternal viral suppression before, during and after breastfeeding. The quantitative cohort thus comprised breastfeeding women, and our sample for qualitative research was drawn from the larger cohort.
